# Disease-Attenuated Pneumococcal Biosynthesis Gene Mutants Invade the Mucosal Epithelium and Induce Innate Immunity

**DOI:** 10.1093/infdis/jiag124

**Published:** 2026-03-24

**Authors:** Caroline M Weight, Gabriele Pollara, Modupeh Betts, Roberta Ragazzini, Elisa Ramos-Sevillano, Jesús Reiné, Matthew Whelan, José Afonso Guerra-Assunção, Michael Connor, Paola Bonfanti, Clare Jolly, Mahdad Noursadeghi, Daniela M Ferreira, Jeremy S Brown, Robert S Heyderman

**Affiliations:** Research Department of Infection, Division of Infection and Immunity, University College London, London, United Kingdom; Research Department of Infection, Division of Infection and Immunity, University College London, London, United Kingdom; Research Department of Infection, Division of Infection and Immunity, University College London, London, United Kingdom; Research Department of Infection, Division of Infection and Immunity, University College London, London, United Kingdom; Epithelial Stem Cell Biology and Regenerative Medicine, Francis Crick Institute, London, United Kingdom; Department of Respiratory Medicine, University College London, London, United Kingdom; Oxford Vaccine Group, University of Oxford, Oxford, United Kingdom; Department of Clinical Sciences, Liverpool School of Tropical Medicine, Liverpool, United Kingdom; Research Department of Infection, Division of Infection and Immunity, University College London, London, United Kingdom; Research Department of Infection, Division of Infection and Immunity, University College London, London, United Kingdom; Chromatin and Infection, Institut Pasteur, Paris, France; Research Department of Infection, Division of Infection and Immunity, University College London, London, United Kingdom; Epithelial Stem Cell Biology and Regenerative Medicine, Francis Crick Institute, London, United Kingdom; Research Department of Infection, Division of Infection and Immunity, University College London, London, United Kingdom; Research Department of Infection, Division of Infection and Immunity, University College London, London, United Kingdom; Oxford Vaccine Group, University of Oxford, Oxford, United Kingdom; Department of Clinical Sciences, Liverpool School of Tropical Medicine, Liverpool, United Kingdom; Department of Respiratory Medicine, University College London, London, United Kingdom; Research Department of Infection, Division of Infection and Immunity, University College London, London, United Kingdom

**Keywords:** biosynthesis genes, host–pathogen interactions, innate immunity, *Streptococcus pneumoniae*, airway epithelium

## Abstract

**Background:**

Nasopharyngeal colonization by *Streptococcus pneumoniae* is characterized by bacterial adherence to epithelial cells, microinvasion, and innate immune activation. Previously, we have shown that two serotype 6B *S pneumoniae* mutant strains affecting bacterial metabolism (*ΔproABC/pia* and *Δfhs/pia*) colonize humans and mice, but in a murine disease model do not cause invasive infection.

**Methods:**

Using an experimental human pneumococcal challenge model, ex vivo airway cells, and in vitro nasopharyngeal epithelium, we explore whether microinvasion and innate immune responses persist despite disease attenuation.

**Results:**

We show that under serum stress, these biosynthesis gene mutations had a broad but different impact on pneumococcal virulence gene expression, oxidative stress regulation, and purine and carbohydrate metabolism genes. However, although these mutations did not attenuate microinvasion in human challenge and epithelial models, there was less transmigration of Detroit 562 nasopharyngeal epithelial cells by the mutants compared to wild-type. Cellular reorganization of primary human airway epithelium varied considerably between strains. Compared to wild-type, infection of Detroit 562 epithelial cells by the *Δfhs/piaA* strain, but not the *ΔproABC/piaA* strain, was less proinflammatory, induced less caspase 8 production, and was associated with increased pneumococcal hydrogen peroxide and reduced pneumolysin secretion.

**Conclusions:**

These findings suggest that differences in microinvasion and epithelial responses were driven by the differential expression of multiple bacterial virulence and metabolic pathways. These data highlight the complex impact of single gene mutations on bacterial virulence and suggest that the virulence determinants of pneumococcal epithelial colonization, microinvasion, and innate immunity are not necessarily directly linked to disease.

## Teaser

Mutations in *Streptococcus pneumoniae* biosynthesis genes had impacts on virulence, oxidative stress, and metabolism. Mutants retained their microinvasive ability but attenuated in transmigration across epithelium, associated with differential epithelial responses. Findings support the hypothesis that multiple pneumococcal factors drive colonization.

## Graphical Abstract

**Graphical Abstract:**
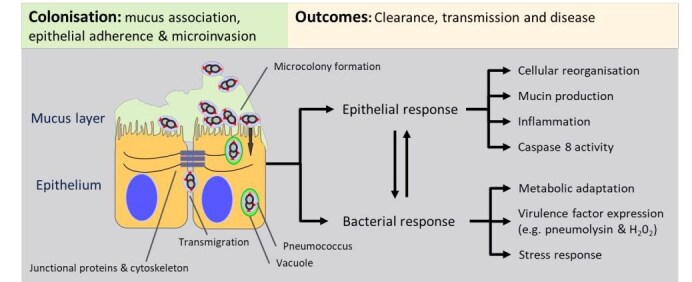
*S. pneumoniae* colonisation is characterised by mucus association, epithelial adherence, microcolony formation and microinvasion - where the pneumococcus invades the epithelial barrier without causing disease. Although mutations in *S. pneumoniae* biosynthesis genes (Δ*proABC* and Δ*fhs*) attenuate disease in a murine model, they do not attenuate microinvasion in either experimental human pneumococcal challenge (EHPC), *ex vivo* or *in vitro* epithelial cells. Transmigration of the epithelial barrier is attenuated. These mutations show straindependent effects on both the epithelial and bacterial responses to infection. Factors such as epithelial cellular reorganisation, inflammation and caspase 8 activity alongside pneumococcal metabolic adaptation, virulence factor expression and response to stress are important components of these processes.

*Streptococcus pneumoniae* is a common commensal of the upper respiratory tract, yet translocation of the pneumococcus to the lungs, blood, or the brain leads to life-threatening disease [1]. Among children aged <5 years, *S pneumoniae* is the leading cause of pneumonia-related deaths globally [2].

We have previously shown that *S pneumoniae* colonization is characterized by epithelial surface microcolony formation and microinvasion [3]. Pneumococcal colonization elicits an innate immune response that may facilitate clearance while also promoting bacterial transmission [3, 4]. Beyond the capsule, numerous virulence factors have evolved to optimize colonization and transmission, rather to cause invasive disease. We hypothesize that epithelial microinvasion and the innate immune response to colonization are not necessarily linked to disease causation.

To test this, we have used two *S pneumoniae* serotype 6B biosynthesis gene mutants (*ΔproABC* and *Δfhs*), which were designed to induce protective immunity through nasopharyngeal colonization without causing disease [5]. The *proABC* operon encodes enzymes for proline biosynthesis, while *fhs* encodes a tetrahydrofolate reductase involved in one-carbon metabolism, both essential for pneumococcal growth and virulence [6–8]. In experimental human pneumococcal carriage (EHPC), *ΔproABC* and *Δfhs* mutants carrying an additional *piaA* deletion (disrupting a key iron uptake transporter, introduced to prevent reversion) retained the ability to colonize and elicit IgG-mediated immunity [9, 10]. These isogenic mutants thus provide a controlled system to investigate epithelial microinvasion and innate immune activation independently of capsular serotype.

Here, we show that although the *ΔproABC/piaA* and *Δfhs/piaA* mutations attenuate disease through broad-ranging impacts on pneumococcal virulence, oxidative stress and metabolism, and reduced epithelial transmigration, they retained the capacity to colonize and invade epithelial cells. The *Δfhs* but not the *ΔproABC* mutant was less proinflammatory. Hence, pneumococcal microinvasion and immune activation are not necessarily precursors to disease progression, highlighting the complex impact of biosynthesis genes on pneumococcal virulence, colonization, and disease.

## MATERIALS AND METHODS

### Bacteria

*Streptococcus pneumoniae* serotype 6B (strain BHN 418 [11]) was a kind gift from Prof. Birgitta Henriques-Normark (Karolinska Institute). *ΔproABC/piaA* and *Δfhs/piaA* mutant strains were generated using overlap extension polymerase chain reaction (PCR) as described by Ramos-Sevillano et al [5]. Aliquot stocks frozen at 0.3 optical density at 600 nm were centrifuged at 8000*g* for 8 minutes and resuspended in 1% fetal bovine serum (FBS) in minimum essential medium (MEM) (Gibco) for infections. Starting inoculums were not significantly different across strains.

### Bacterial RNA Sequencing Sample Collection, Sequencing, and Reads

Pneumococcal strains were grown to mid log phase and pellets resuspended in THY or undiluted human serum for 60 minutes. Cultures were pelleted and stored in RNA Protect at −70°C [8]. Total RNA from each library was ribo-depleted and sent for Illumina Next Seq Sequencing (Pathogen Genomics Unit, University College London [UCL]) as detailed in the Supplementary Information.

### RNA Samples and Sequencing

Confluent monolayers of Detroit 562 cells were infected for 4 hours before collection in RNALater. RNA was extracted using a Qiagen RNAEasy micro kit and treated for DNA removal using a Qiagen Turbo DNA-free kit. Quality was assessed and quantified using a BioAnalyzer (Agilent 2100). Samples were sequenced using Illumina NextSeq 500/550 High Output 75 cycle kit giving 15–20 million 41 bp paired-end reads per sample (Pathogen Genomics Unit, UCL).

### Differential Gene Expression Analyses

The generated raw count matrix was imported into R software (version 3.4.2) and normalization of counts and differential gene expression (DGE) analysis was performed using the package SARTools, a DESeq2 R pipeline, using a false discovery rate (FDR) of 0.05 [12, 13] and categorized as differentially expressed. Pathway analysis of DGEs was performed using the Reactome database via the Database for Annotation, Visualization, and Integrated Discovery (DAVID; https://davidbioinformatics.nih.gov/home.jsp), limiting significant pathways to those with FDR *q* < .05). The expression of transcriptional modules was derived from geometric mean expression of all constituent genes within a module as previously described [14, 15], using Mann–Whitney tests to determine significant differences in module expression between experimental conditions. Ingenuity pathway analysis (Qiagen) was used to predict upstream kinases and transcription factors that regulate the expression of genes, with significant regulators determined by *P* values <.01 [15]. These results were visualized as a network diagram using the R package igraph (https://r.igraph.org/).

### EHPC Model

A full explanation of experimental design and selection criteria has been previously described [3], and details can be found in the Supplemental Information.

### Normal Human Bronchial Epithelial Airway Cells

Normal human bronchial epithelial airway (NHBE-A) cells (ATCC PCS-300-010) were cultured as described in the Supplemental Information.

### Human Epithelial Cell Lines

Human pharyngeal carcinoma Detroit 562 epithelial cells (ATCC CCL-138) were cultured in 10% FBS in α-MEM media (Gibco). For each set of experiments, cells were used within 5 passages. Cells consistently tested PCR negative for *Mycoplasma* (Cambridge Biosciences).

### Pneumococcal–Epithelial Cell Co-culture

For association and internalization assays, confluent monolayers of Detroit 562 cells cultured on 12 well plates (Corning), were infected (1 cell to 10 pneumococci) for 3 hours in 1% fetal calf serum–MEM or 6 hours for NHBE-A cells in 1% Pneumocult media without supplements [3]. Cells were washed 3 times in HBSS with calcium and magnesium before incubation in 1% saponin for 10 minutes at 37°C. Bacterial dilutions were plated on horse blood agar for 16 hours and colony-forming units (CFUs) counted. For internalization quantification, 200 µg/mL gentamicin was added for 1 hour before incubation with saponin.

For transmigration assays, confluent monolayers of Detroit 562 cells cultured on 3-µm pore PET Transwell Inserts (Thermo Fisher) [3] were infected for 3 hours. Fifty microliters of basal media was repeatedly removed to measure bacterial load by counting CFU/well.

### Confocal Microscopy

Epithelial monolayers on transwell membranes were infected, then fixed in 4% Paraformaldehyde (Pierce, methanol free) as previously described [3] and detailed in the Supplementary Information. Cells were processed for image analysis using an inverted Zeiss LSM (either 700 or 880). Z stacks were recorded at 1-µm intervals at either 40× oil or 63× oil objectives. Cells and bacteria were segmented and analyzed using CellProfiler automated image analysis package for Python [16]. Nuclei were segmented using a primary object segmentation algorithm using the thresholded DAPI intensity. Cellular periphery segmentation was carried out through use of secondary object tentpole algorithm using thresholded actin of cellmask intensities. Bacteria were segmented as intracellular puncta using primary object “spot” segmentation using thresholded bacterial channel fluorescence. Cells object masks associated with bacteria puncta objects were classified as positive and the remaining population was classified as negative. For each respective protein of interest, integrated intensity measurements were quantified within the area of the cell mask of each cell population.

### Electron Microscopy

Preparation of Detroit 562 cells and NHBE-A cells was performed as previously reported [3] and described in the Supplemental Information.

### Pneumolysin Activity

Adapted from Kirkham et al [17]. Two percent horse blood (EO Labs) in phenol-free α-MEM (Life Technologies) in 96 U-bottom plates (Corning) was mixed with 2.5 × 10^6^ per well of whole bacterial suspensions. Solutions were incubated for 30 minutes at 37°C and additionally centrifuged at 1000*g* for 1 minute. Absorbance from supernatant was recorded at 540 nm on a Tecan plate reader.

### Hydrogen Peroxide Assay

Detroit 562 cells cultured in white 96-well plates (Corning) were infected and incubated with H_2_O_2_ substrate according to manufacturer’s instructions (ROS-Glo H_2_O_2_ Assay, Promega). Luminescence was read using a GloMax Multi Detection System plate reader (Promega).

### Caspase Assay

Detroit 562 cells were cultured in white 96-well plates (Corning) and infected. Samples were incubated with Caspase-Glo 1 Reagent, Caspase-Glo 1 YVAD-CHO Reagent, Caspase-Glo Z-VAD-FMK Reagent, or Caspase-Glo 8 Reagent for 1 hour and according to manufacturer’s instructions (Promega). Luminescence was read using a GloMax-Multi Detection System plate reader (Promega).

### Statistical Analysis

Conditions within each experiment were performed in duplicate or triplicate, and each experiment was performed independently >3 times, unless stated otherwise. Error bars represent standard error of the mean, unless stated otherwise. GraphPad Prism version 7 was used for parametric (t-tests or analysis of variance) or nonparametric (Mann–Whitney or Kruskal–Wallis tests) analysis, which was based on the Shapiro–Wilk normality test. Ad hoc tests were performed using Tukey (for parametric data) or Dunn (for nonparametric data) multiple comparisons test. *P* values <.05 were considered significant.

### Data and Code Availability

Source data are provided as a source data file. RNA sequencing (RNA-seq) data from the Detroit 562 cell infections are publicly accessible through Zenodo (https://doi.org/10.5281/zenodo.7997789).

## RESULTS

### Deletion of the *proABC* and *fhs* Genes Have Broad-Ranging Differential Effects on Pneumococcal Virulence Gene Expression and Metabolic Gene Pathways When Under Stress

We have previously shown that *Δ*fhs and *Δ*proABC showed considerable derangement in global gene transcription when cultured in human serum compared to growth under optimal nutrient conditions [8]. Metabolic analysis suggested that in sera, *Δfhs* had impaired stringent responses, and both mutants were under increased oxidative stress and had altered lipid profiles [8]. The *ΔproABC* mutation resulted in the accumulation of glycolytic pathway and peptidoglycan synthesis intermediates. To further understand the link between disease attenuation, epithelial colonization, and innate immune responses, we extended the transcriptomic analysis of the response of the mutants to serum stress [8]. This revealed upregulation of virulence factors such as *ply*, *nanA*, and *psaA*; those regulating oxidative stress like *SpxB*, *lctO*, and *adhE*; and genes involved in purine and carbohydrate metabolism (Supplementary Table 1). Phosphotransferase systems, amino nucleotide sugar, fructose and mannose, and metabolic pathways were the most significantly enriched pathways in the *ΔproABC* mutant (Table 1). In contrast, competence, purine, pyruvate, metabolic pathways, amino nucleotide sugar pathways, and secondary metabolites were the most significantly enriched pathways in the *Δfhs* mutant under stress (Table 1).

**Table 1.** Differentially Expressed Pathways From Single Isogenic Mutants in Human Serum Compared to Wild-Type

Pathway	Gene Ratio	Adjusted *P* Value	*q* Value
*Δfhs* vs wild-type			
Fatty acid metabolism/biosynthesis	10/57	1.08E-09	6.49E-10
Biosynthesis of secondary metabolites	26/57	2.09E-04	1.26E-04
Purine metabolism	10/57	1.34E-03	8.06E-04
Quorum sensing	11/57	3.20E-03	1.92E-03
Pantothenate and CoA biosynthesis	5/57	3.59E-03	2.16E-03
β-lactam resistance	5/57	7.59E-03	4.57E-03
Propanoate metabolism	4/57	1.51E-02	9.09E-03
2-oxocarboxylic acid metabolism	4/57	1.51E-02	9.09E-03
Metabolic pathways	40/57	1.94E-02	1.16E-02
Amino sugar and nucleotide sugar metabolism	7/57	4.22E-02	2.54E-02
Pyruvate metabolism	5/57	4.31E-02	2.59E-02
*ΔproABC* vs wild-type			
Fatty acid metabolism/biosynthesis	10/71	1.17E-08	8.94E-09
Galactose metabolism	12/71	1.25E-04	2.47E-08
Phosphotransferase system	13/71	2.54E-03	1.93E-03
Amino sugar and nucleotide sugar metabolism	9/71	2.89E-02	2.20E-02

Differentially expressed Kyoto Encyclopedia of Genes and Genomes (KEGG) pathways identified in single isogenic mutants grown in human serum compared to the wild-type strain. “GeneRatio” indicates the proportion of differentially expressed genes (DEGs) associated with each pathway (eg, 10/57 denotes that 10 of the 57 DEGs in the *Δfhs* mutant were annotated to the fatty acid metabolism/biosynthesis pathway). Adjusted *P* values and *q* values represent multiple-testing corrected significance levels.

### 6B Disease–Attenuated Mutants Colonize and Microinvade Nasal Epithelium in EHPC

**Figure 1.**
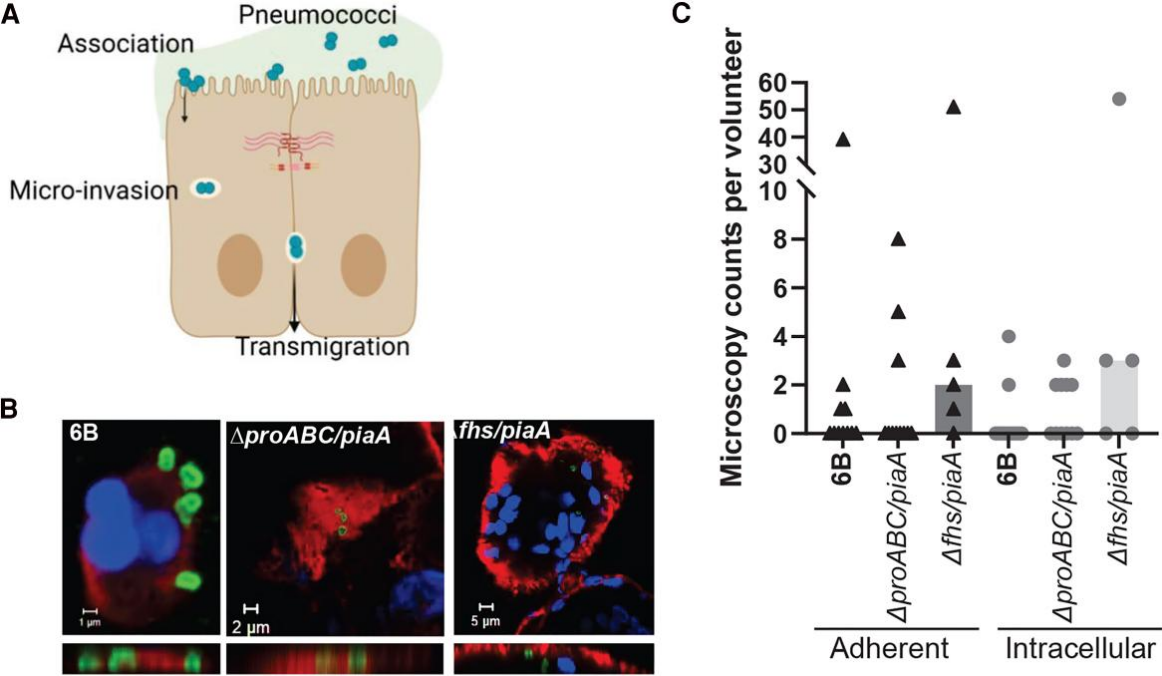
Pneumococcal–epithelial interactions in the experimental human pneumococcal carriage model. *A*, Schematic showing that during colonization, pneumococci adhere to the epithelial cell surface (association) and internalize inside epithelial cells (microinvasion). Created in BioRender. Weight, C. (2026) https://BioRender.com/td6yhit. Mucus on apical membrane of epithelial cells, adjoined by cellular junctions; diplococci pneumococci; intracellular vacuole containing pneumococci. *B*, Representative microscopy images showing pneumococcal–epithelial interactions by the 6B wild-type or *ΔproABC/piaA* or *Δfhs/piaA* mutants. Surface carbohydrates highlight cell surface, nuclei stained with DAPI and pneumococci visible. *C*, Microscopy counts for each inoculated strain 6 days postchallenge per volunteer. Adherent represents pneumococcal surface association and intracellular represents pneumococci that have microinvaded (inside) epithelial cells.

The EHPC model is well established for studying pneumococcal carriage and disease pathogenesis [3, 10, 18]. Mucus flow, antimicrobial peptides, antibody, competition with the resident microbiota, and epithelial turnover limit pneumococcal–epithelial interactions. Using nasal curette samples from EHPC, colonization was found to peak at day 6 postinoculation [3]. We therefore investigated whether the *ΔproABC/piaA* or *Δfhs/piaA* mutations affected epithelial association and microinvasion (Figure 1*A*). We showed colonization by the WT and mutant strains at day 6 postchallenge (Figure 1*B*): 4 of 11 volunteers challenged with the WT, 5 of 11 volunteers with the *ΔproABC/piaA*, *and* 4 of 5 volunteers with the *Δfhs/piaA*. Microinvasion (intracellular bacteria) was seen in 2 of 11 volunteers with the WT, 5 of 11 volunteers with the *ΔproABC/piaA,* and 3 of 5 volunteers with the *Δfhs/piaA* (Figure 1*C*, Supplementary Table 2). Together, these data show that compared to WT, there was no attenuation of colonization or microinvasion with either the *ΔproABC/piaA* or *Δfhs/piaA* strains in EHPC.

### Colonization and Microinvasion of Human Airway Epithelium by the Disease-Attenuated Mutants Is Associated With Cellular Reorganization and Preservation of Barrier Function

**Figure 2.**
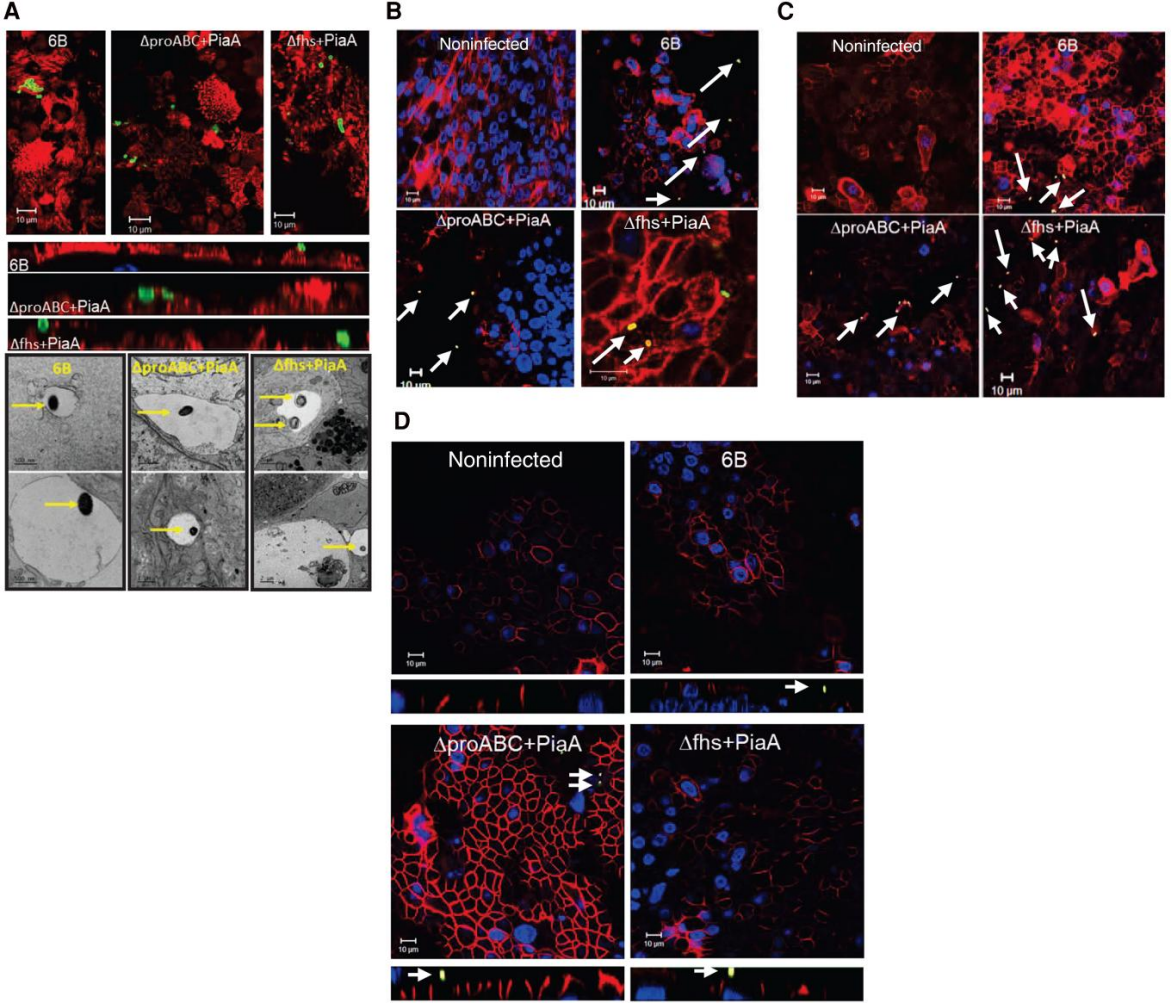
Impact of pneumococcal infection on normal human bronchial epithelial airway (NHBE-A) cell junctional protein expression and barrier integrity. NHBE-A cells were cultured at air-liquid interface and infected with pneumococcal strains for 6 hours. *A*, Top: Representative confocal microscopy images from 4 independent experiments with replicates, showing pneumococcal association with the epithelium (surface carbohydrates and nuclei visible) at 6 hours postinfection. Bottom: Representative transmission electron microscopy images from 2 independent experiments, showing pneumococcal microinvasion examples of each strain (arrows), enclosed within a vacuole after a 4-hour infection. Representative confocal microscopy images from 4 independent experiments with replicates showing pneumococcal (indicated with arrows) interactions with cellular junctional proteins; JAM-A (*B*), ZO-1 (*C*), and β-catenin (*D*) in primary human bronchial epithelial airway cells after a 6-hour exposure. Nuclei stained with DAPI. Co-localization of junctional proteins and pneumococci are highlighted with arrows.

To explore whether disease attenuation influences epithelial cellular organization, we infected NHBE-A cells. We observed epithelial association with all 3 strains (Figure 2*A*, top). Transmission Electron Microscopy demonstrated that intracellular bacteria for all 3 strains were likely enclosed within vacuoles (Figure 2*A*, bottom). We next assessed barrier integrity and pneumococcal co-localization with junctional proteins. WT and mutant strains co-localized with junctional adhesion molecule A (JAM-A; Figure 2*B*, arrows), with no change in JAM-A expression. Zonula occludens 1 (ZO-1) expression was increased with WT (Figure 2*C*) at the junctional membrane. The intensity of β-catenin staining significantly increased following direct interaction (“pneumococci associated positive”; Supplementary Figure 1*A*) with either *ΔproABC/piaA* or *Δfhs/piaA*, compared to WT 6B (Figure 2*D*). β-catenin expression significantly increased across the epithelium in cells exposed to *ΔproABC/piaA* strain, regardless of direct interactions or noninfected (“pneumococcal associated negative”; Supplementary Figure 1*A*). Overall, these changes suggested cellular reorganization following pneumococcal exposure without affecting barrier integrity (Supplementary Figure 1*B*) or cell viability (Supplementary Figure 1*C*).

**Figure 3.**
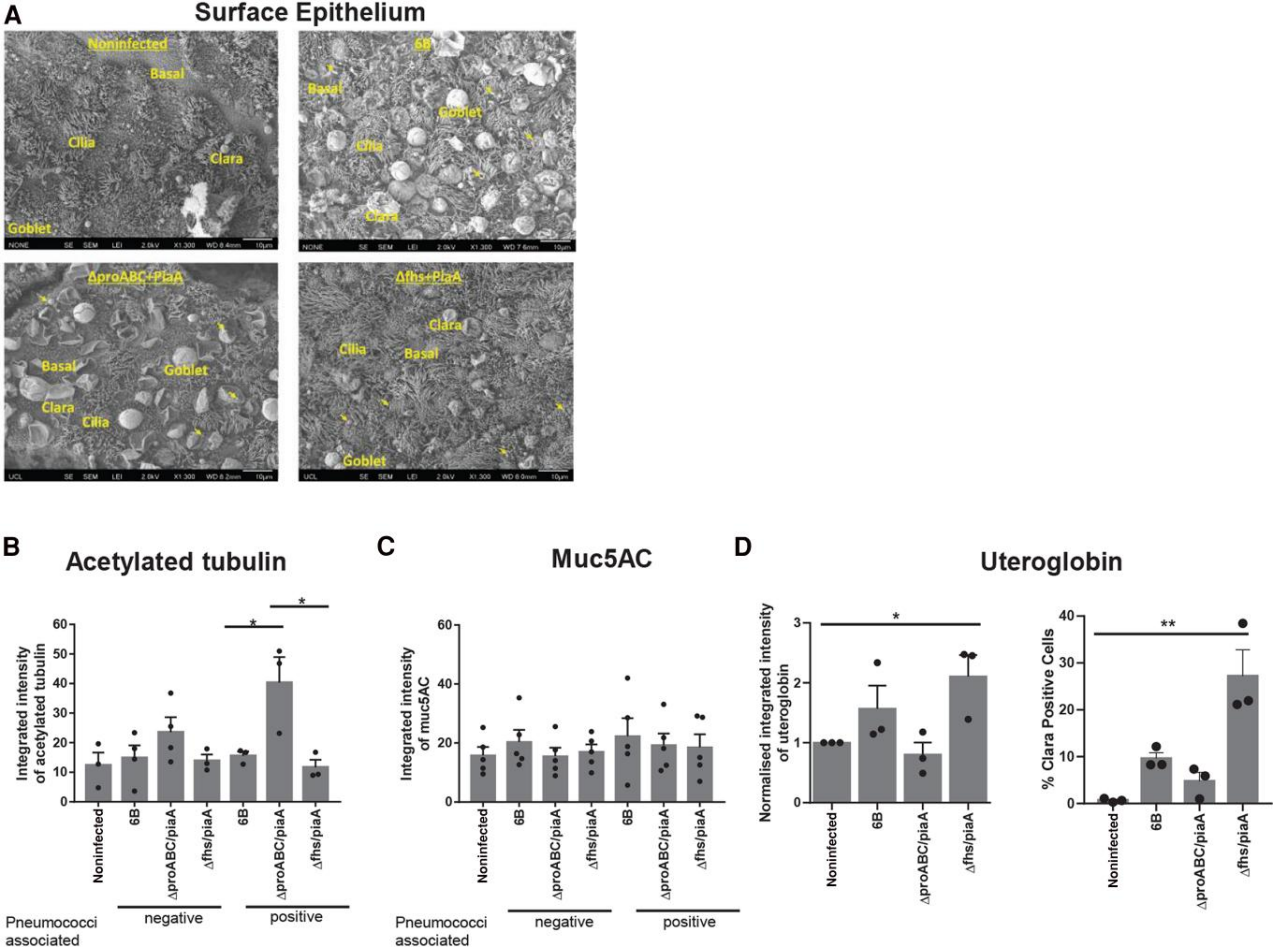
Pneumococcal–epithelial interactions with normal human bronchial epithelial airway (NHBE-A) cells. Cells were cultured at air-liquid interface and infected with pneumococcal strains for 6 hours. *A*, Representative scanning electron microscopy images from 2 independent experiments, showing the surface of the epithelium. Representative examples of differentiated epithelia (basal cells, Clara cells, goblet cells, ciliated cells) are shown in text. Colonizing pneumococci are highlighted with arrows. Integrated intensity of acetylated tubulin (*B*) or Muc5ac (*C*) in pneumococcal negative or positive cells from 2 or more images. Acetylated tubulin: negative, *P* = .2677; positive, *P = .017 (analysis of variance). Mucin5ac: negative, *P* = .6852; positive, *P* = .8510; n = 3 or 4 independent experiments. *D*, Integrated intensity of uteroglobin normalized to noninfected cells (left, *P = .0115 noninfected vs infected, Kruskal–Wallis) and percentage of Clara-positive cells following infection (right, **P = .0003 noninfected vs strains, **P = .0036 6B vs mutant strains, Kruskal–Wallis). n = 3 independent experiments.

NHBE cells cultured at an air-liquid interface differentiate into a pseudostratified columnar ciliated epithelium, consisting of goblet, Clara, basal, and ciliated cells (Figure 3*A*). Following infection, strains were observed among cilia (acetylated tubulin–positive cells; Supplementary Figures 2*A* and 3) and the *ΔproABC/piaA* co-localized with mucus (mucin5ac positive, arrows; Supplementary Figure 2B). Infection with the *ΔproABC/piaA* strain increased expression of acetylated tubulin within pneumococcal-associated cells (Figure 3*B*), known to be associated with increased microtubule conformation and elasticity [19, 20]. Infection with WT and *ΔproABC/piaA* but not *Δfhs/piaA* resulted in an increased abundance of secreted mucus globules compared to uninfected cells (Figure 3*A*). This appeared due to secretion of preformed mucus rather than de novo production, as expression of Mucin5AC was unaffected (Figure 3*C*, Supplementary Figure 2B). In contrast, only infection with *Δfhs/piaA* caused increased expression of uteroglobin, a multifunctional epithelial secreted protein marker for Clara cells with anti-inflammatory and anti-chemotactic properties (Figure 3*D*, Supplementary Figure 2C).

### *Δfhs/piaA* but Not the *ΔproABC/piaA* Mutations Attenuate the Proinflammatory Epithelial Innate Response to Pneumococcal Colonization and Microinvasion

**Figure 4.**
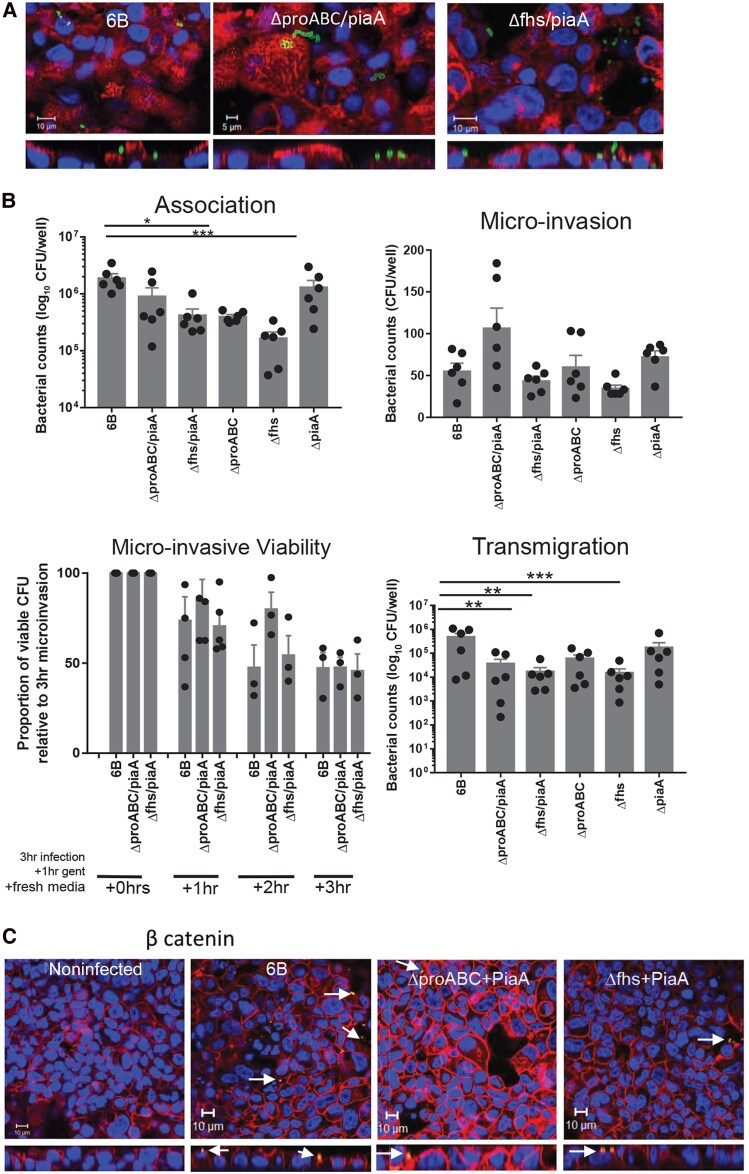
Strain association, microinvasion, and transmigration of Detroit 562 cells. Cells were infected with *Streptococcus pneumoniae* for 3 hours and colony-forming unit (CFU) counts recorded. *A*, Representative confocal microscopy images from 6 independent experiments with replicates, showing pneumococcal interactions with cell surface carbohydrates and nuclei stained with DAPI. *B* (top left), Association of pneumococcal strains to epithelial cells. ***P = .0009 (Kruskal–Wallis), n = 6 with replicates. *B* (top right), Internalization of strains into cells, following a gentamicin protection assay. *P = .0219 (Kruskal–Wallis), n = 6 with replicates. *B* (bottom left), Pneumococcal viability inside epithelial cells following a gentamicin protection assay. n = 3 or 4 independent experiments with replicates. Counts were normalized to microinvasion counts for each strain after 3 hour of infection and compared to 6B wild-type; +1 hour, *P* = .6517; +2 hours, *P* = .1377; +3 hours, *P* = .1183 (analysis of variance). *B* (bottom right), Transmigration of pneumococcal strains across confluent monolayers on transwell inserts. CFUs were recorded from the basal chamber. n = 6 independent experiments with replicates. ***P < .001, **P < .0.01 (Kruskal–Wallis). *C*, Representative confocal microscopy images from 6 independent experiments with replicates, showing pneumococcal interactions with cellular β-catenin and nuclei. Nuclear DAPI stain (blue).

We have shown that Detroit 562 epithelial nasopharyngeal cells represent a good model for responses to *S pneumoniae* [3] (Supplementary Figure 4). Indeed, infection of Detroit 562 cells showed that WT *ΔproABC/piaA* and *Δfhs/piaA* strains formed colonies on the epithelial surface (Figure 4*A*), with limited differences between these strains (Figure 4*B*, top left). Microinvasion was observed with all strains (Figure 4*B*, top right) and showed no differences in pneumococcal intracellular viability (Figure 4*B*, bottom left) or replication (Supplementary Figure 5*A–F*). In line with attenuation of disease, the *ΔproABC/piaA* and *Δfhs/piaA* were impaired in their ability to transmigrate across the epithelium (Figure 4*B*, bottom right). Importantly, the single *Δ*p*iaA* mutation in the *S pneumoniae* serotype 6B did not affect epithelial association, microinvasion, and transmigration, linking the differences seen to the *Δfhs* and *ΔproABC* mutations (Figure 4*B*). Epithelial β-catenin expression was also affected following infection with the *ΔproABC/piaA* and *Δfhs/piaA* strains, compared to the WT in pneumococcal-associated and nonassociated cells (Figure 4*C*).

**Figure 5.**
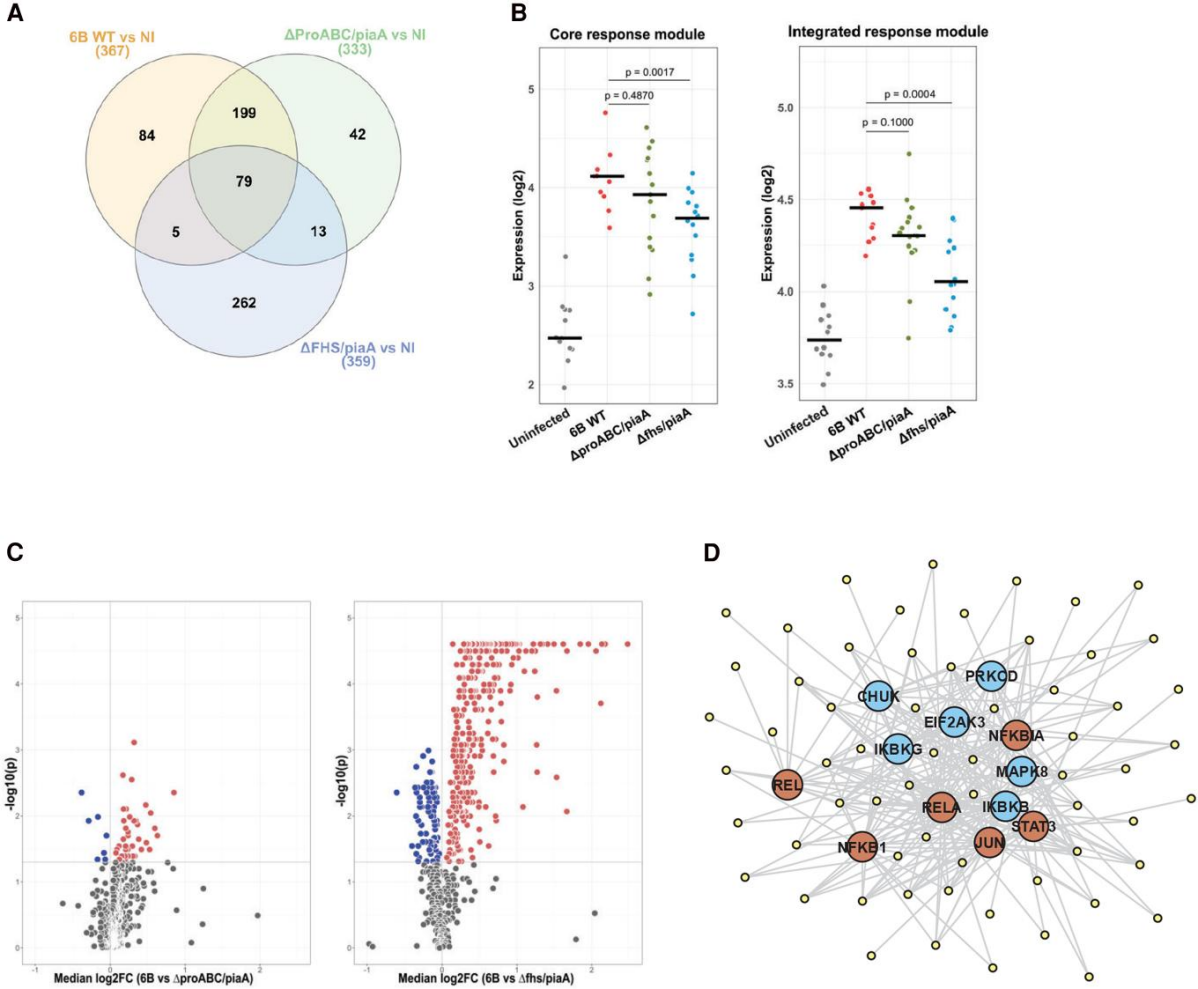
Detroit 562 cell transcriptomic responses to pneumococcal strain infections. *A*, Venn diagram representing genes with significantly elevated expression (log_2_ fold change [FC] >0, false discovery rate <0.05) following infection with pneumococcal strains relative to noninfected (NI) cells. Numbers on Venn diagram segments reflect gene expression shared and unique between strains. *B*, Expression of core or integrated response modules in epithelial cell transcriptomes following infection. *P* values derived by unpaired Mann–Whitney tests between specified groups. *C*, Volcano plots depicting constituent genes of integrated response module, comparing expression between infection with 6B wild-type (WT) and *ΔproABC/piaA* (left panel) or *Δfhs/piaA* (right panel). Significant difference defined by log_2_FC >0 and *P* > .05 by Mann–Whitney test. *D*, Network plot describing predicted relationship between genes with elevated expression following 6B infection than after *Δfhs/piaA* infection (dashed region in (*C*) and the 6 most significantly enriched upstream kinases (CHUK, PRKCD, EIF2AK3, IKBKG, MAPK8, IKBKB) and transcription factors (REL, RELA, NFKB1A, NFKB1, JUN, STAT3). Experimental data were derived from uninfected (n = 12), 6B-infected (n = 11), *ΔproABC/piaA*-infected (n = 17), and *Δfhs/piaA*-infected (n = 14) samples from 6 independent experiments.

We then explored how epithelial transcriptional responses relate to strain colonization and microinvasion. Infection with all strains resulted in increased expression of 79 genes, a “core response” module (Figure 5*A*, Supplementary Tables 4–7), mapping to induction of inflammatory signaling pathways (Supplementary Table 8). Strain differences were also evident, with responses induced by *ΔproABC/piaA* strain resembling those seen with 6B WT, in contrast to qualitatively different, but relatively attenuated, responses seen with *Δfhs/piaA* strain (Figure 5*A*, Supplementary Tables 4–7). We next analyzed the core response module, and the expression of all 684 upregulated genes induced from all strains as an “integrated response” module (Supplementary Table 7). Both modules revealed markedly diminished responses with the *Δfhs/piaA* strain compared to WT (Figure 5*B*). Assessing the expression of individual genes within the integrated gene list yielded similar observations, with minimal differences in genes induced by WT and *ΔproABC/piaA* strains, whereas *Δfhs/piaA* demonstrated attenuated induction of 322 genes compared to WT (Figure 5*C*, Supplementary Table 9). To explore further the host response not induced by the *Δfhs/piaA* mutant, we focused on predicted upstream regulators of these genes, revealing that *Δfhs/piaA* induced lower activity of intracellular kinases (CHUK [IKKα], IKBKB [IKKβ], IKBKG [NEMO]) and transcription factors (RELA [p65], NFKB1 [p50], REL [c-Rel], NFKBIA [Iκ-Bα]) that are predominantly associated with activation of the NF-κB pathway (Figure 5*D*).

### Enhanced Epithelial Caspase 8 Activity Following Infection With WT and *ΔproABC/piaA* Strains

To further explore epithelial innate immune response to colonization, we assessed differential caspase activation. Caspase activity indicates that a cell is undergoing programmed cell death (eg, apoptosis, pyroptosis, or PANoptosis) or represents immune cell recruitment through the release of cytokines and promotes NF-κB signaling that we observed in Figure 5 [21]. Ultimately, the NLRP3 inflammasome drives programmed epithelial cell death via caspase 1, caspase 3/7, and caspase 8 activity [22].

**Figure 6.**
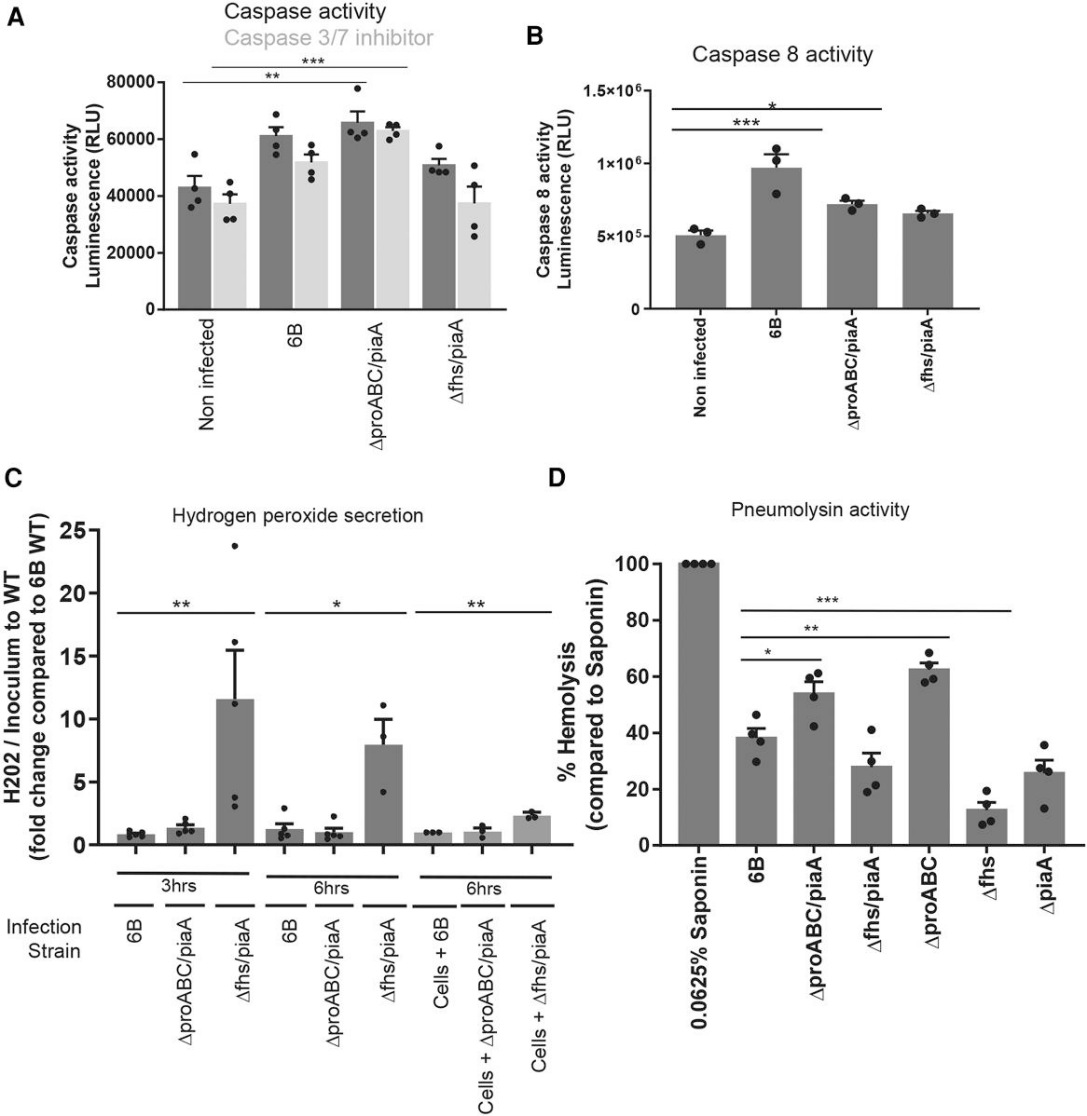
Detroit 562 cell and pneumococcal responses following colonization. Caspase activity was assessed by luminescence, following infection with pneumococcal strains for 6 hours. *A*, Caspase 3/7 activity was determined following incubation with the specific inhibitor Z-VAD-FMK. n = 4 with replicates. In comparison to noninfected cells, caspase 3/7 **P = .0022; caspase 3/7 + Z-VAD-FMK inhibitor ***P = .0005. In comparison to 6B wild-type (WT): caspase 3/7, *P = .0215; caspase 3/7 + Z-VAD-FMK inhibitor, **P = .0012 (Kruskal–Wallis). *B*, Caspase 8 activity was assessed following infection with pneumococcal strains. n = 3 with replicates. In comparison to noninfected cells, ***P = .0015 (ANOVA) and, in comparison to 6B WT, *P = .0172 (analysis of variance [ANOVA]). *C*, Pneumococcal fold change of hydrogen peroxide production from the mutants compared to WT, normalized to the colony-forming unit (CFU) counts. Dark bars: 3 hours, **P = .0087 (ANOVA); 6 hours, *P = .0209 (Kruskal–Wallis). Light bars represent fold change of hydrogen peroxide production from the mutants compared to WT, normalized to the CFU counts, following infection of Detroit 562 cells. Media was replaced after 1 hour of infection and cells were incubated for a further 5 hours. n = 3 independent experiments with replicates. Compared to 6B WT, **P = .0034 (ANOVA). *D*, Pneumolysin activity was assessed using a red blood cell lysis assay. Strains were incubated with red blood cells for 30 minutes and absorbance measured. n = 4 experiments with replicates. Pneumococcal strain hemolytic activity is calculated in comparison to 100% lysis by 0.0625% saponin. ***P < .0001 compared to 6B WT (ANOVA).

Compared to noninfected cells, all strains increased epithelial caspase activity 6-hour postinfection, although this was only significant following infection with *ΔproABC/piaA* (Figure 6*A*, dark gray bars). Incubating infected cell cultures with either a caspase 1 inhibitor (YVAD-CHO) or caspase 3/7 inhibitor (Z-VAD-FMK), showed that increased caspase activation following infection with WT and *Δfhs/piaA* was not due to caspase 1 activity (Supplementary Figure 6). Compared to noninfected cells, caspase 3/7 accounted for 3% and 15% of the caspase activity induced following infection with WT and *Δfhs/piaA*, respectively (Figure 6*A*). In contrast, *ΔproABC/piaA* strain did not induce caspase 3/7 activity. Caspase 8 activation was significantly increased following epithelial infection with 6B WT and *ΔproABC/piaA* strain (Figure 6*B*).

### *Fhs* Gene Mutation Is Associated With Increased Pneumococcal Hydrogen Peroxide Secretion and Decreased Pneumolysin Activity

We postulated that differences in cellular reorganization, the innate inflammatory response and caspase 8 activation seen between *ΔproABC/piaA* and *Δfhs/piaA* mutants could be explained by differential expression of pneumococcal H_2_O_2,_ which has been suggested as an adaptive mechanism for colonization [23]. As LctO was transcriptionally upregulated in both mutants compared to WT during serum stress (Supplementary Table 1), we assessed whether pneumococcal H_2_O_2_ production was also affected. After 3 and 6 hours, we detected significantly higher levels of H_2_O_2_ in *Δfhs/piaA* cultures compared to 6B WT and *ΔproABC/piaA* strains (Figure 6*C*, left dark bars). Furthermore, this was maintained during epithelial cell contact (Figure 6*C*, right light bars).

Pneumolysin can both activate and suppress the host innate immune response and mediates the hemolytic activity characteristic of the pneumococcus [24–26]. H_2_O_2_ increases the release of pneumolysin but paradoxically also negatively impairs its hemolytic activity [27]. In line with this paradoxical effect, the higher production of H_2_O_2_ by *Δfhs/piaA* strain was associated with increased *ply* gene expression but reduced red blood cell lysis compared to 6B WT and *ΔproABC/PiaA* (Figure 6*D*).

## DISCUSSION

Here, we show preservation of epithelial microinvasion when using isogenic serotype 6B biosynthesis pathway mutant strains, which are attenuated for systemic disease but not carriage [5, 8, 28]. Epithelial barrier transmigration was significantly less with the *Δfhs/piaA* and *ΔproABC/piaA* mutations compared to WT. Importantly, while epithelial microinvasion, cellular reorganization, the innate epithelial immune response, and the propensity to cause invasive disease following pneumococcal colonization are all strain-dependent, no single determinant explained these differences.

In our NHBE-A cell infection model [29], infection with WT and mutant strains resulted in epithelial cellular reorganization involving β catenin, acetylated tubulin, and uteroglobin. However, strain differences with the impact on junctional or cytoskeletal protein organization or mucus production did not explain the disease attenuation seen with *Δfhs/piaA* and *ΔproABC/piaA*.

Detroit 562 cell infection with all 3 strains induced gene expression reflecting innate inflammatory pathway activity typical of pneumococcal infection [3], including several cytokines and chemokines. A notable observation was the attenuated induction of these responses following *Δfhs/piaA* strain infection, in particular, reduced activation of signaling pathways leading to induction of NF-κB–regulated genes. Moreover, the host response to *Δfhs/piaA* also initiated a distinct prosurvival and adaptation profile, including genes like JUN, SMARCA4, NFAT5, KLF6, and TCF4, which are involved in homeostatic signaling and chromatin remodeling [30, 31]. This suggests that infection with *Δfhs/piaA* strain results in a more tolerant epithelial response and supports our hypothesis that colonization occurs without overt disease. Ultimately, these capsule-independent differences in pneumococcal–epithelial interactions may shape the differences seen in adaptive immune responses identified using EHPC [18, 32].

Importantly, while all strains adhered and microinvade the epithelium, WT serotype 6B infection led to pneumococcal transmigration, a more proinflammatory innate immune transcriptomic profile, and increased caspase 8 activity, indicative of a robust cellular response to *S pneumoniae*, with *ΔproABC/piaA* and *Δfhs/piaA* exhibiting attenuation of many of these responses. This suggests that pneumococcal sensing by and transmigration across the epithelium is affected by disruption of *S pneumoniae* metabolism [25, 33]. Pneumolysin is a key driver of epithelial inflammatory responses and increased acetylated tubulin and microtubule stabilization [3, 19]. Under stress conditions, the *ΔproABC* and *Δfhs* mutations upregulated multiple metabolic pathways, presumably as a compensatory mechanism, and also genes encoding several pneumococcal virulence factor genes (see graphical abstract summary) including pneumolysin and H_2_O_2_. Based on these findings, we propose that differential expression of pneumolysin and H_2_O_2_ by the WT and mutant strains may contribute to the distinct patterns of epithelial responses that we observed, with the potential to influence both bacterial clearance and transmission [1]. Whether the distinct epithelial innate immune response to *Δfhs/piaA* infection is due to diminished NF-κB and MAPK activation (potentially linked to extracellular receptor engagement), reduced caspase 8 activity mediated by enhanced pneumococcal H_2_O_2_ release, decreased pneumolysin activity, or a compensatory metabolic response to the stress remains to be determined. As seen in “professional” intracellular bacterial species such as *Listeria* and *Salmonella*, the host response to microinvasion might also promote pneumococcal transmission [34, 35]. Whether the transmissibility of the less proinflammatory *Δfhs/piaA* is affected could be explored further using EHPC [36].

In conclusion, these data further support our proposed paradigm that epithelial microinvasion by *S pneumoniae* is frequent but does not necessarily lead to disease. Microinvasion may aid transmission through immune evasion and localized inflammation and transmigration across the epithelium may represent the first step toward transition to invasive disease. Our findings also highlight the broad-ranging effects of single gene mutations in pneumococcal biosynthesis pathways, which may explain the complexity of differences between seemingly closely related strains. The interplay between *S pneumoniae* and the host epithelium is modulated by key mediators such as hydrogen peroxide and pneumolysin, which can have both enhancing and inhibitory effects on epithelial immune responses depending on infection context. However, strain differences in subsequent epithelial cell microinvasion, cellular reorganization, and inflammatory responses appear driven by the differential expression of multiple bacterial virulence and metabolic pathways, rather than any single determinant.

## Supplementary Material

jiag124_Supplementary_Data

## Supplementary Data

Supplementary materials are available at The Journal of Infectious Diseases online (http://jid.oxfordjournals.org/). Supplementary materials consist of data provided by the author that are published to benefit the reader. The posted materials are not copyedited. The contents of all supplementary data are the sole responsibility of the authors. Questions or messages regarding errors should be addressed to the author.
