# Supplementary material for: Disease-Attenuated Pneumococcal Biosynthesis Gene Mutants Invade the Mucosal Epithelium and Induce Innate Immunity
Source: J Infect Dis. 2026 Mar 24;233(5):e1141–53. doi: 10.1093/infdis/jiag124 (PMC13175632; doi:10.1093/infdis/jiag124)

Disease-attenuated pneumococcal biosynthesis gene mutants invade the mucosal epithelium and induce innate immunity

Weight *et al*

SUPPLEMENTAL INFORMATION

SUPPLEMENTAL METHODS AND PROTOCOLS

Bacterial RNA-seq sample collection, sequencing and reads

Mutant pneumococcal strains (three biological replicates for each mutant) and 6B WT (three biological replicates) were grown in THY to mid log phase and the pellets resuspended in THY and undiluted human serum for 60mins. The culture was then pelleted, and RNA stored in RNA Protect at -70°C before extracting total RNA [1]. Total RNA from each library was ribo-depleted and sent for Illumina Next Seq Sequencing Pathogen Genomics Unit (PGU), UCL. The quality of raw FASTQ reads was checked by FastQC v0.11.5, Babraham Bioinformatics, UK and visualised using multiQC v1.9 [2]. Reads were trimmed using Trimmomatic 0.39 [3]. Trimmed reads were also checked by FastQC and multiQC. Trimmed reads were then mapped to KEGG annotated 6B genome sequence (670-6B, Accession: CP002176.1) using bowtie2 with default settings [4]. Conversion into BAM files was performed using SAMtools [5]. Mapped reads were visualized in the Integrated Genome Browser [6]. FeatureCounts v2.0.0 was used to summarize read counts for each annotated feature in the respective reference genomes in multimapping mode (-M) [7].

Experimental Human Pneumococcal Carriage Model (EHPC)

A full explanation of experimental design and selection criteria has been previously described [8]. Following written informed consent, healthy non-smoking adults between the ages of 18 – 59 were inoculated with 80,000 CFU/nostril live strains of *S. pneumoniae*. Mucosal cells from

the inferior turbinate using a plastic Rhino-probe™ (curettage, Arlington Scientific) were collected prior to inoculation, and days 2 and 6 post-inoculation. Samples were processed for confocal microscopy analysis. Nasal wash samples were collected for CFU count analysis. There were no adverse events. A positive carrier was defined as colonisation at any time point (day 2 of day 6 post inoculation) as detected by culture [8]. Ethical approval was given by NHS Research and Ethics Committee (REC)/Liverpool School of Tropical Medicine (LSTM) REC, reference number: 18/NW/0481 and Human Tissue Authority licensing number 12548.

Normal Human Bronchial Epithelial Cells (NHBE-A)

NHBE-A cells (ATCC® PCS-300-010™) were plated at $6x10^3$ cells/cm^2 on a layer of sub-lethally γ-irradiated (60 Gy) mouse embryonic fibroblasts 3T3-J2 cells (kind donation from Howard Green, Harvard Medical School, Boston [9]) with keratinocyte culture medium cFAD (3:1 DMEM (Gibco) to Ham F-12 Nut Mix (Gibco), 10% FBS (HyClone, Sigma), 1% Penicillin-Streptomycin (100X, Sigma), 0.4 µg/mL Hydrocortisone (Calbiochem, USA), 5 µg/ml Insulin (various), 10-10 Cholera Toxin (Sigma) and 2x10-9 Triodothyronine (Sigma)). Epithelial cells were stimulated with 10 ng/mL human epidermal growth factor (hEGF, PeproTech, USA) on days 3 and 5. Cultures were grown at 37^oC 6% CO_2. For NHBE-A differentiation in Air Liquid Interface (ALI) culture, cells were plated at $6x10^4$/cm^2 on 1µm pore PET cell culture inserts (12 wells, Greiner). Chambers were supplemented with cFAD medium. After 48 hours, medium was removed from the top chamber. Lower chamber media was replaced with PneumaCult™ differentiation Medium (Stemcell Technologies) every 2 days for 3 weeks. Antibiotics were removed from media and cells were washed 24 hours before experiments.

Confocal Microscopy

Sample processing was performed as previously described [8]. Briefly, mucosal cells from the EHPC model were placed directly into 4% PFA and cytospun onto microscope slides. NHBE-

A cells and Detroit 562 cells cultured on transwell membranes were fixed in 4% PFA (Pierce, Methanol Free). Cells were permeabilised with 0.2% Triton X-100 for 10 minutes, blocked for one hour in blocking buffer (3% goat serum and 3% BSA in PBS (Sigma)) and incubated with pneumococcal antisera Pool Q (raised against the polysaccharide capsule, SSI Diagnostica) for one hour. Wheat germ agglutinin conjugated to Rhodamine (Vector Labs) was added along with goat-anti rabbit IgG conjugated to Alexa Fluor 488 for 45 minutes in blocking buffer. Primary (used at 1:100) antibodies used for epithelial cell proteins can be found in Supplemental Information Table 2. Secondary (used at 1:500) antibodies were either goat anti-mouse or goat anti-rabbit Alexa Fluor 555 or 546, ThermoFisher. Of note, for epithelial protein antibodies raised in rabbit (Uteroglobin), no pneumococcal staining was possible and therefore pneumococci were not visible on the images. Primary and secondary antibodies to epithelial cell proteins were incubated with pneumococcal samples alone as negative controls. Secondary antibody-only cell samples were used as negative controls for confirmation that primary antibodies do not cross-react and that background non-specific staining of secondary antibodies is negligible. DAPI was added for 5 minutes and cells were mounted with Aqua PolyMount (VWR International) and a coverslip on the microslide. Pneumococci were recorded by manual scanning of the whole cytospin for the EHPC samples. More than three fields of view were recorded for each bacterial strain for Detroit 562 cell infections, using inverted Zeiss LSM (either 700 or 880) confocal microscopes. Images were processed with Zeiss LSM Image Browser. Z stacks were recorded at 1µm intervals at either 40x oil or 63x oil objectives to determine pneumococcal surface adherence, through co-localisation with WheatGerm Agglutinin or host proteins, or, internalised through proximity to the cell nucleus, underneath the layer of surface carbohydrates (Supplementary Fig 2). Automated image analysis was carried out using Cellprofiler [10]. Each channel was pre-screened in Fiji ImageJ in which the intensity distribution histogram was visually analysed to determine that saturated pixels were not overly represented in each channel.

Electron Microscopy

Preparation of Detroit 562 cells and NHBE-A cells was identical and previously described [8]. Briefly, cells cultured on transwells were fixed with 2% paraformaldehyde and 1.5% glutaraldehyde in 0.1 M cacodylate buffer and post-fixed in 1% OsO4 / 1.5% K4Fe(CN)6 in 0.1 M cacodylate buffer pH7.3. For scanning electron microscopy, samples were dehydrated in graded ethanol-water series and infiltrated with Agar 100 resin mix. Ultra-thin sections were cut at 70-80 nm using a diamond knife on a Reichert ultracut microtome. Sections were collected on 300 mesh copper grids and stained with lead citrate. For transmission electron microscopy the samples were hardened at 60°C for 48 hours. The coverslip was removed, and a representative area was selected. Ultra-thin sections were cut at 70-80 nm using a diamond knife on a Reichert ultra-cut S microtome. Sections were collected on copper grids and stained with lead citrate. Samples were viewed with a Joel 1010 transition electron microscope and the images recorded using a Gatan Orius CCD camera.

TABLE AND FIGURE LEGENDS

Table 1. Differentially upregulated genes by *S. pneumoniae* biosynthesis mutants in comparison WT under serum stress. Enrichment analysis of virulence genes, experimentally deleted biosynthesis genes, purine related genes, hydrogen peroxide regulators and carbohydrate metabolism. A comparison of differential gene expression was assessed when pneumococcal strains were cultured in human serum, comparing the single ∆*proABC* ∆*fhs* mutant relative to the WT strain.

Table 2. Microscopy and microbiology counts for each inoculated strain 6 days post challenge per volunteer. Adherent represents pneumococcal surface association and intracellular represents pneumococci that have micro-invaded (inside) epithelial cells.

Table 3. Primary antibodies used for Microscopy. Primary and secondary antibodies for epithelial proteins. Primary antibodies were added for 1 hr at room temperature before washing off in HBSS and adding secondary antibodies for 45 minutes.

Figure 1. Impact of pneumococcal infection on NHBE-A cell junctional protein expression and barrier integrity. (A) Integrated intensity of β catenin in pneumococcal negative (not pneumococcal associated) or positive (pneumococcal associated) cells. Non infected v pneumococcal negative cells **** p <0.0001 (NI v Δ*proABC/piaA*), ** p = 0.0026 (NI v Δ*fhs/piaA*); non-infected v pneumococcal positive cells; **** p <0.0001 (NI v Δ*proABC/piaA* and NI v Δ*fhs/piaA*); Kruskal-Wallis, n = 4 independent experiments. (B) Transepithelial electrical resistance (TEER) was recorded pre- and post- 6hr infection to assess barrier integrity. N = 3 independent experiments with replicates. Average TEER pre-infection was 466Ω.cm^2 (430-484 Ω.cm^2, p = 0.1458 ANOVA) and average TEER post-infection was 545Ω.cm^2 (527-567 Ω.cm^2. p = 0.04744 ANOVA). Change in TEER comparing non-infected cells to infected cells p = 0.6592, Kruskal-Wallis. Error bars represent SEM. (C) After 6hrs infection, supernatant was collected and analysed for lactate dehydrogenase secretion from the apical and basal chamber. Non-infected v infected; p >0.05, ANOVA. N = 5-6 independent experiments with replicates.

Figure 2. Pneumococcal – epithelial interactions with NHBE-A cells. Representative confocal microscopy images from 4 independent experiments with replicates for (A) Ciliated cells (acetylated tubulin, red), (B) Goblet cells (mucin5ac, red, (arrows indicate co-localisation of mucin5ac with pneumococci)), (C) Clara cells (uteroglobin, red) after 6hr infection. Pneumococci (green), nuclei (DAPI, blue), colocalization of red and green appears as yellow.

Figure 3. Ciliated NHBE-A cells. Representative example of pneumococcal (green) associations with ciliated (acetylated tubulin, red) cells as illustrated with the Δ*proABC*+*PiaA* mutant in NHBE-A cells.

Figure 4. Surface adherence and microinvasion. Representative examples of pneumococci (green) associations with epithelial cells (red, surface carbohydrate staining). (A) Adherence of 6B WT on surface of NHBE-A cells, z-stack view indicated with arrow. (B) Examples of surface adherence in Detroit cells with 6B WT (top) and Δ*fhs*+*PiaA* (bottom). (C) Examples of microinvasion in Detroit cells with Δ*proABC*+*PiaA* (top) and Δ*fhs*+*PiaA* (bottom), underneath the level of the apical epithelial surface.

Figure 5. Association, microinvasion and transmigration of pneumococci following 1hr infection in Detroit 562 cells. (A-D) Pneumococcal-epithelial interactions; association, micro-invasion and transmigration of pneumococcal strains after 1hr infection in Detroit 562 cells and CFUs were recorded. (A) Association of 6B strains. $p = 0.0024$. (B) Internalisation of 6B strains. $p = 0.0107$. (C) Transmigration of 6B strains. Pneumococcal strains were added to the apical chamber of a transwell insert with a confluent monolayer of Detroit 562 cells. CFUs were recorded over time from the basal chamber, reflecting bacteria that had transmigrated across the epithelium. $p = 0.0106$. (D) Pre ($p = 0.5403$) and post ($p = 0.6384$) inoculum. N = 4 independent experiments with replicates (Kruskal-Wallis). (E) Pre and post inoculum after 3 hours infection. Pre- (light grey) and post- (dark grey) inoculums after 3 hours incubation with epithelial cells. Pre- $p = 0.8366$, Post- $p = 0.0160$, 6B v isogenic mutants (Kruskal-Wallis), n = 6 with replicates. (F) Cell supernatant from infected cells was analysed for lactate dehydrogenase secretion. N = 3 - 5 with replicates. $p = 0.046$ compared to non-infected cells, and $p = 0.5649$ between 6B WT and mutant strains (ANOVA). (G) Epithelial cell permeability was assessed via leak to 4kDa FITC-Dextran into the basal chamber. N = 4 with replicates. *p*

<0.0001 compared to non-infected cells, and *p* = 0.0689 between 6B WT and mutant strains (Kruskal-Wallis).

Figure 6. Effects of pneumococcal infection on caspase 1 activity in Detroit 562 cells. Caspase 1 activity was determined following incubation with the specific inhibitor YVAD-CHO. Negative signals indicate caspase activity other than caspase 1. N = 6 with replicates. In comparison to non-infected cells, *p* = 0.39; caspase+YVAD-CHO inhibitor *p* = 0.3604 and, in comparison to 6B WT, *p* = 0.6226; caspase+YVAD-CHO inhibitor *p* = 0.5386 (Kruskal-Wallis).

REFERENCES

1. Ramos-Sevillano E, Ercoli G, Betts M, et al. Essential role of proline synthesis and the one-carbon metabolism pathways for systemic virulence of Streptococcus pneumoniae. mBio **2024**; 15:e0175824.
2. Ewels P, Magnusson M, Lundin S, Kaller M. MultiQC: summarize analysis results for multiple tools and samples in a single report. Bioinformatics **2016**; 32:3047-8.
3. Bolger AM, Lohse M, Usadel B. Trimmomatic: a flexible trimmer for Illumina sequence data. Bioinformatics **2014**; 30:2114-20.
4. Langmead B, Salzberg SL. Fast gapped-read alignment with Bowtie 2. Nat Methods **2012**; 9:357-9.
5. Li H, Handsaker B, Wysoker A, et al. The Sequence Alignment/Map format and SAMtools. Bioinformatics **2009**; 25:2078-9.
6. Nicol JW, Helt GA, Blanchard SG, Jr., Raja A, Loraine AE. The Integrated Genome Browser: free software for distribution and exploration of genome-scale datasets. Bioinformatics **2009**; 25:2730-1.
7. Liao Y, Smyth GK, Shi W. featureCounts: an efficient general purpose program for assigning sequence reads to genomic features. Bioinformatics **2014**; 30:923-30.
8. Weight CM, Venturini C, Pojar S, et al. Microinvasion by Streptococcus pneumoniae induces epithelial innate immunity during colonisation at the human mucosal surface. Nat Commun **2019**; 10:3060.
9. Rheinwald JG, Green H. Serial cultivation of strains of human epidermal keratinocytes: the formation of keratinizing colonies from single cells. Cell **1975**; 6:331-43.
10. Carpenter AE, Jones TR, Lamprecht MR, et al. CellProfiler: image analysis software for identifying and quantifying cell phenotypes. Genome Biol **2006**; 7:R100.

Supplemental Table 1: Differentially upregulated genes by *S. pneumoniae* biosynthesis mutants in comparison WT under serum stress

Gene	Δ*proABC* vs. WT		Δ*fhs* vs. WT	
	FoldChange	P-adjust	FoldChange	P-adjust
Deleted biosynthesis genes				
fhs	1.16	3.30E-03	-12.04	3.10E-54
proA	-11.31	2.39E-37	1.38	1.06E-02
proB	-11.47	8.22E-34	1.35	1.59E-02
proC	-11.47	1.74E-27	1.38	4.84E-02
Virulence Genes				
nanA	7.75	9.88E-20	2.87	1.92E-11
nanB	-1.03	9.24E-01	-1.36	1.39E-01
ply	5.91	6.74E-33	9.22	9.05E-94
psaA	2.93	4.21E-15	3.73	3.81E-41
Purine metabolism				
purN	3.33	1.89E-08	6.40	9.12E-26
SP670_0127	1.99	8.51E-03	2.96	8.95E-07
purH	1.84	3.77E-03	3.81	7.48E-14
purD	2.80	4.33E-08	3.22	6.10E-14
purE	2.23	4.27E-04	2.67	8.14E-07
purK	3.27	6.15E-09	3.61	2.62E-16
purB	2.03	4.67E-05	2.11	2.91E-09
SP670_0123	3.66	2.48E-18	6.96	3.83E-48
Oxidative stress				
spxB	1.12	5.65E-01	1.01	9.43E-01
lctO	3.17	4.16E-13	1.39	1.02E-02
spx	-1.03	9.16E-01	-1.45	4.84E-02
adhE	1.32	1.83E-01	1.31	5.90E-02
dpr	-1.30	0.14	-1.88	5.79E-06
PTS/ Carbohydrate metabolism				
SP670_0137	64.83	1.93E-60	1.69	3.82E-04
SP670_0138	67.98	4.63E-51	2.00	6.16E-04
SP670_0139	43.35	6.09E-44	1.67	1.88E-03
SP670_0140	41.35	1.64E-14	1.84	1.75E-03
SP670_0141	33.44	1.73E-12	1.93	3.36E-04
SP670_0142	22.86	2.26E-10	1.77	8.37E-05
SP670_0700	36.80	1.32E-11	1.98	2.07E-02
SP670_0701	42.56	5.29E-55	1.67	1.40E-02
SP670_0702	34.42	3.07E-50	1.86	2.63E-07
SP670_0703	24.49	1.64E-31	1.64	1.41E-02
SP670_0704	22.50	1.52E-27	2.04	2.73E-05

Supplemental Table 2: Microscopy and microbiology counts for each inoculated strain 6 days post challenge per volunteer

6B	MICROSCOPY		MICROBIOLOGY
	Adherent	Intracellular	
#1	0	0	0
#2	2	0	520
#3	0	0	0
#4	1	0	0
#5	0	0	9
#6	1	2	223
#7	0	0	5725
#8	39	4	1110
#9	0	0	0
#10	0	0	767
#11	0	0	0

Δ*proABC/piaA*	MICROSCOPY		MICROBIOLOGY
	Adherent	Intracellular	
#1	0	0	0
#2	0	0	0
#3	0	0	0
#4	5	3	0
#5	3	2	0
#6	8	2	5
#7	0	0	0
#8	0	2	0
#9	0	2	1
#10	0	0	0
#11	0	0	0

Δ*fhs/piaA*	MICROSCOPY		MICROBIOLOGY
	Adherent	Intracellular	
#1	2	0	21856
#2	1	3	0
#3	3	3	16
#4	51	54	0
#5	0	0	520325

Supplemental Table 3. Primary antibodies used for Microscopy

Protein	Primary antibody
	Species raised, catalogue and clone, company
JAM-A	α-mouse, SC53623, J10.4, Santa Cruz
Claudin 1	α-mouse, 37-4900, 2H10D10, ThermoFisher
Claudin 4	α-mouse, H00001364, MO2, Biotechne
ZO-1	α-mouse, 339100, Invitrogen
β catenin	α-mouse, 2677, L54E2, Cell Signalling Technology
Acetylated tubulin	α-mouse, T7451, 6-11B-1, Merck
Muc5ac	α-mouse, MAB2011, CLH2, Merck
Uterogloblin	α-rabbit, PA5102469, Invitrogen

Supplementary Figure 1. Impact of pneumococcal infection on NHBE-A cell junctional protein expression and barrier integrity.

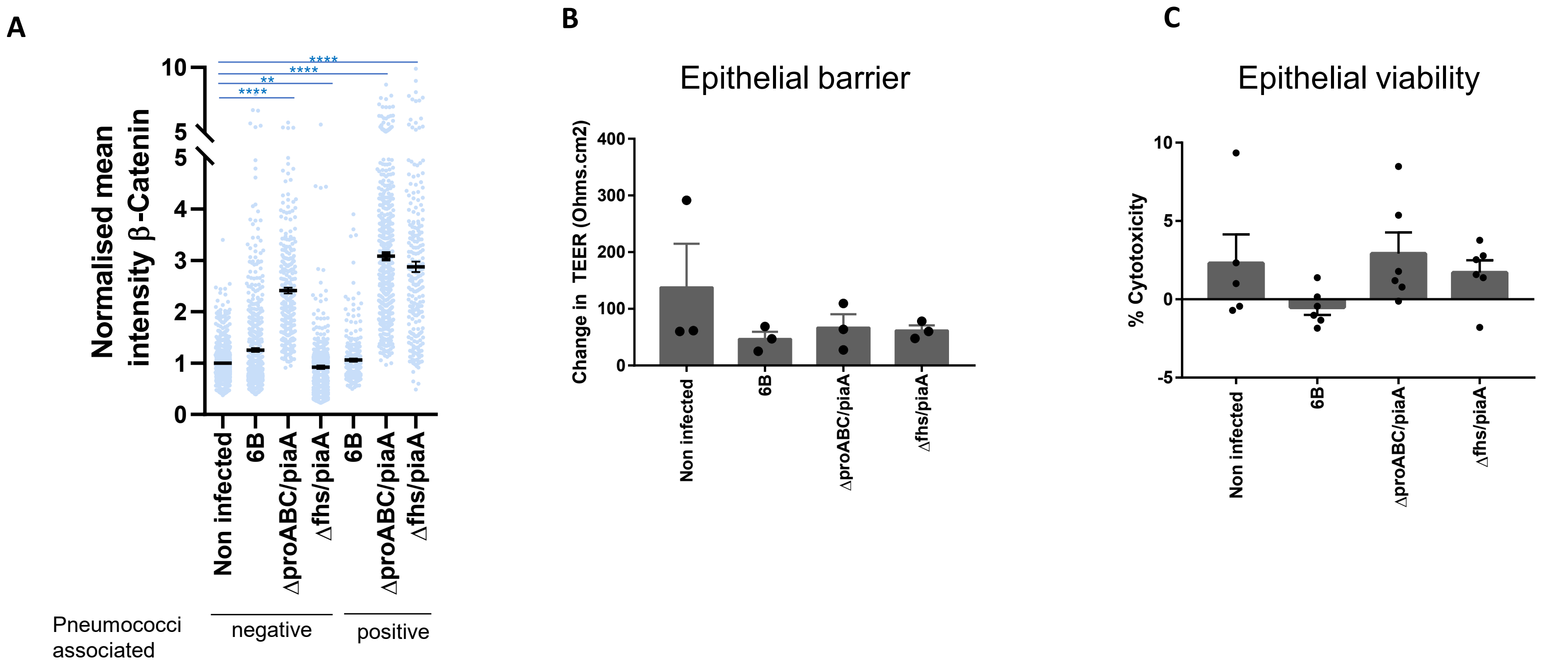

Supplementary Figure 2. Pneumococcal – epithelial interactions with NHBE-A cells

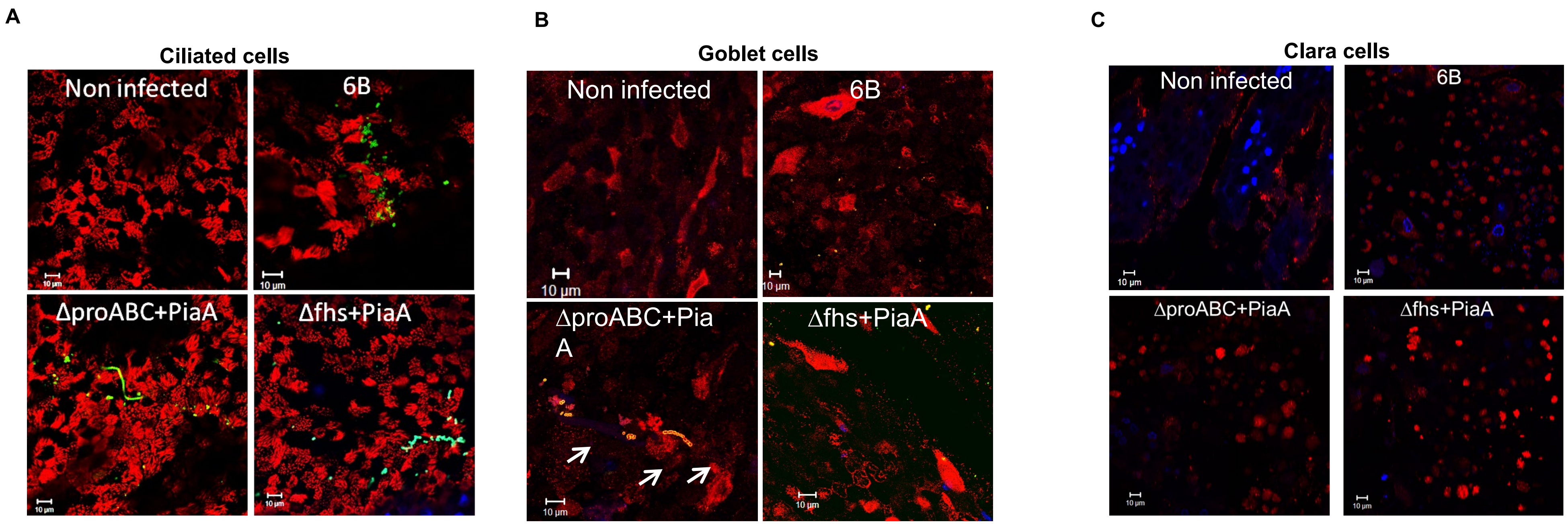

Supplemental Figure 3. Ciliated NHBE-A cells

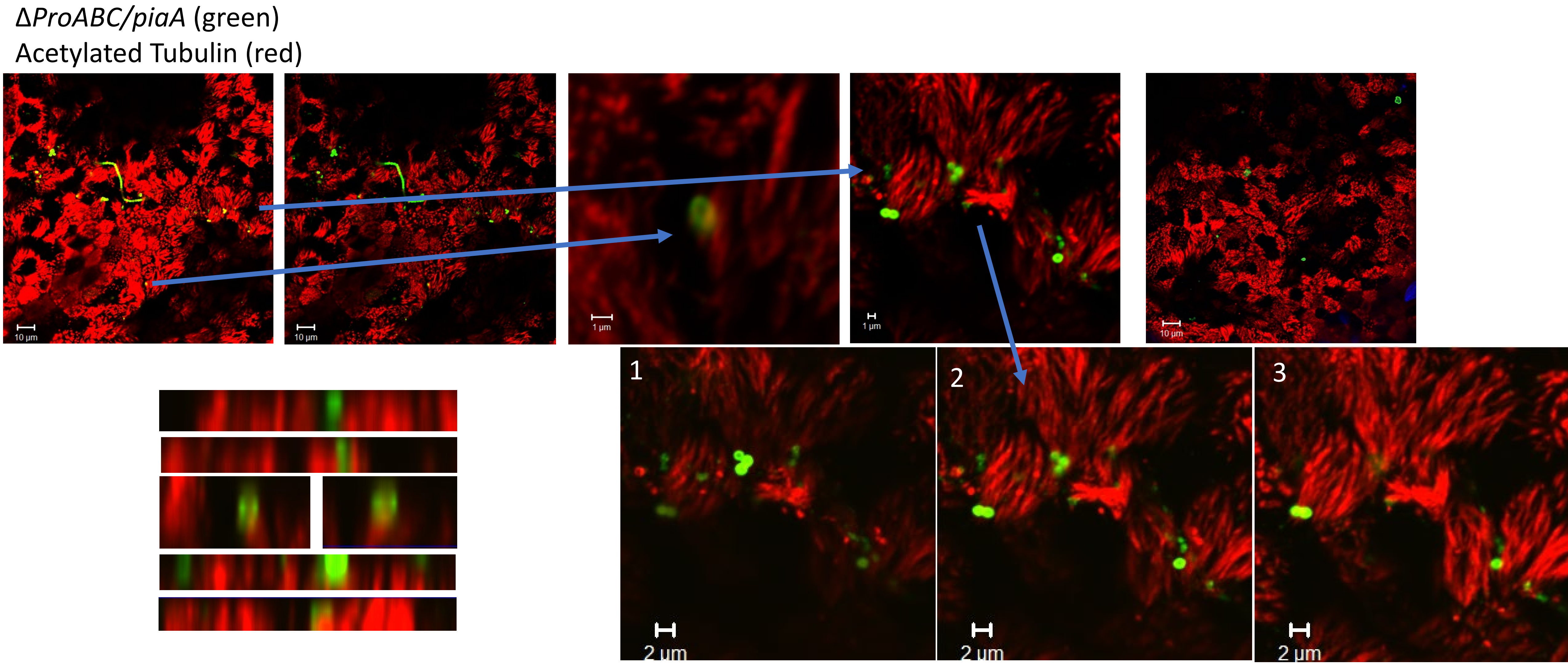

Supplemental Figure 4. Surface adherence and microinvasion

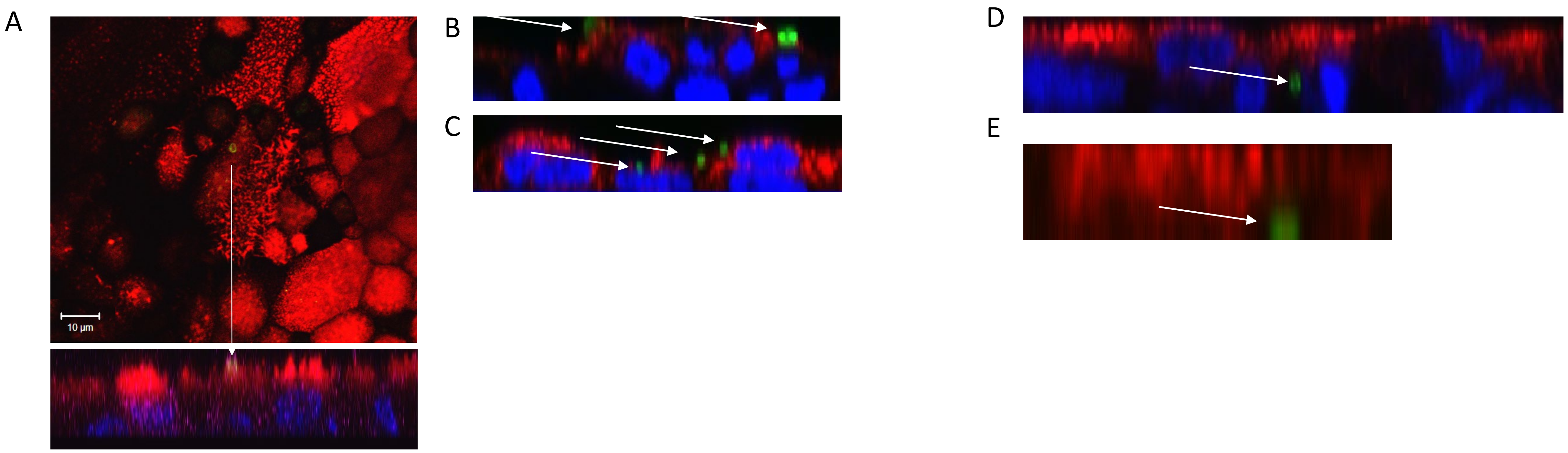

Supplemental Figure 5. Association, microinvasion and transmigration of pneumococci following infection in Detroit 562 cells

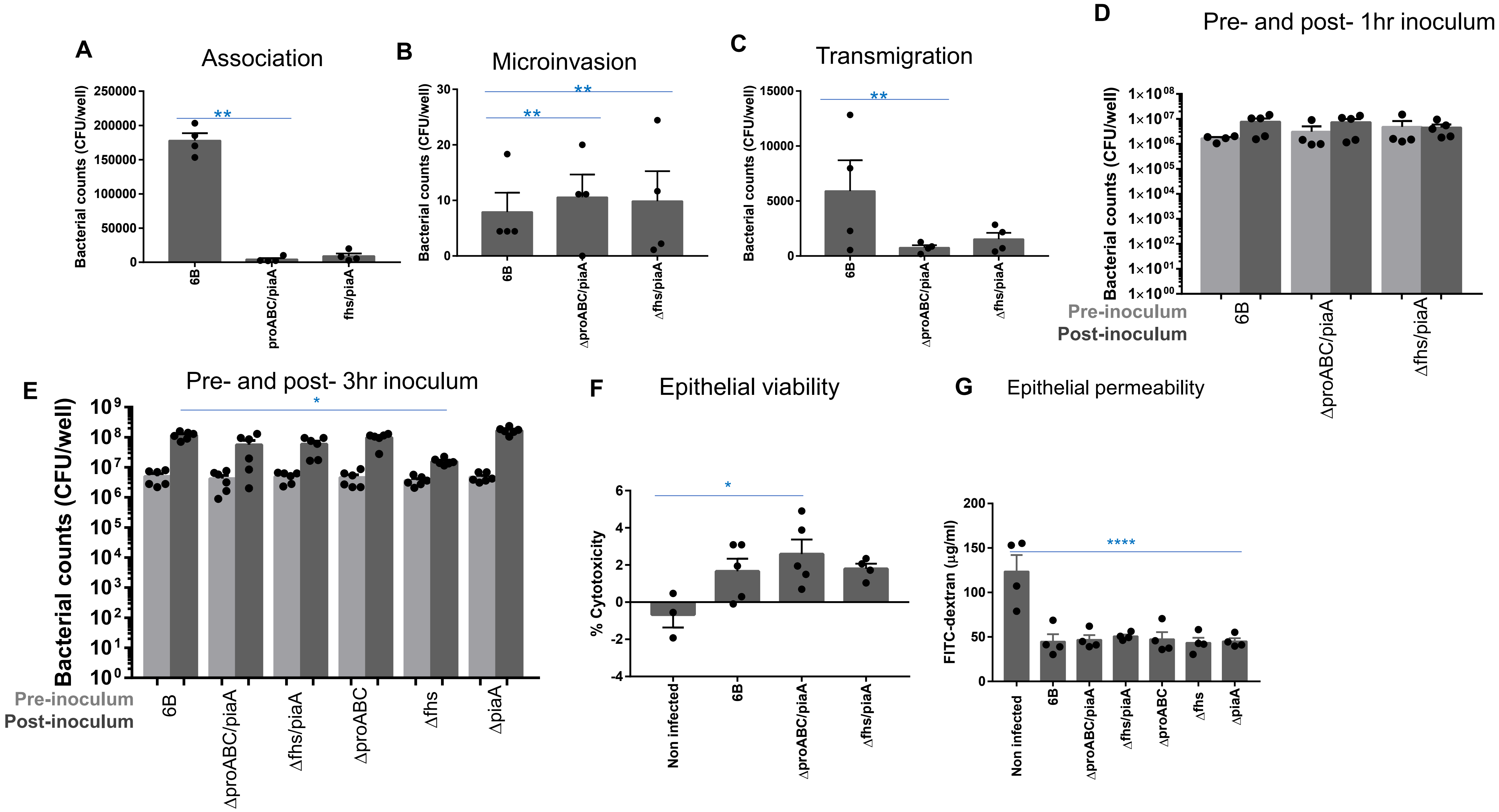

Supplemental Figure 6. Effects of pneumococcal infection on caspase 1 activity in Detroit 562 cells.

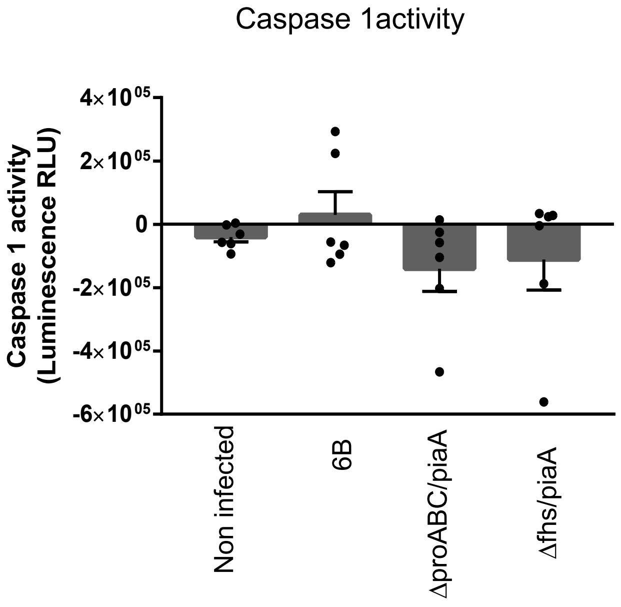

Supplement: jiag124_Supplementary_Data [file jiag124_supplementary_data.zip › SUPPLEMENTAL_INFORMATION_WEIGHT_JID_FINAL.pdf]
